# Supplementary material for: Microbiome composition and function within the Kellet’s whelk perivitelline fluid
Source: Microbiol Spectr. 2024 Feb 9;12(3):e03514-23. doi: 10.1128/spectrum.03514-23 (PMC10913743; doi:10.1128/spectrum.03514-23)
Supplement: Fig. S1-S9 — Supplementary figures. [file spectrum.03514-23-s0001.pdf]

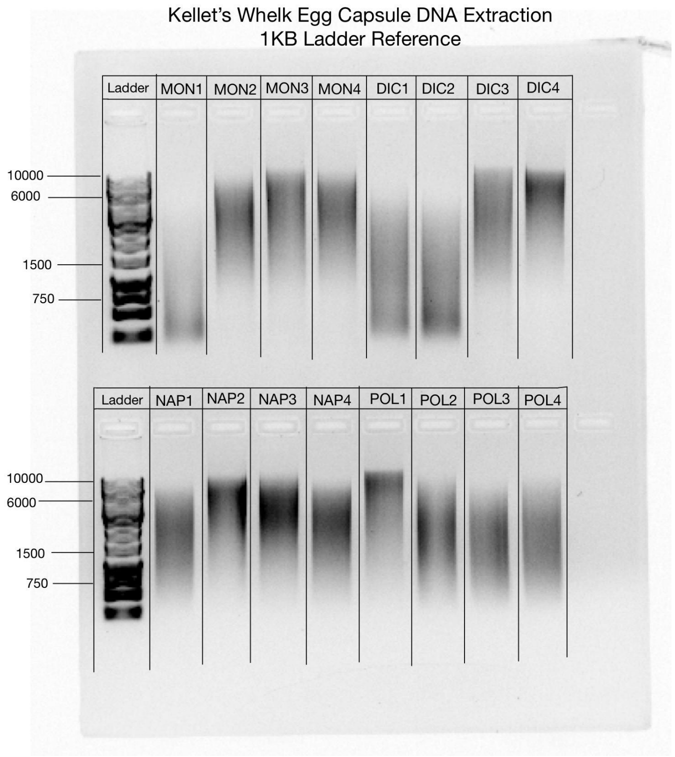

Figure S1. Gel electrophoresis imaging for the experimental PowerSoil DNA extraction samples for 16S rRNA gene sequencing.

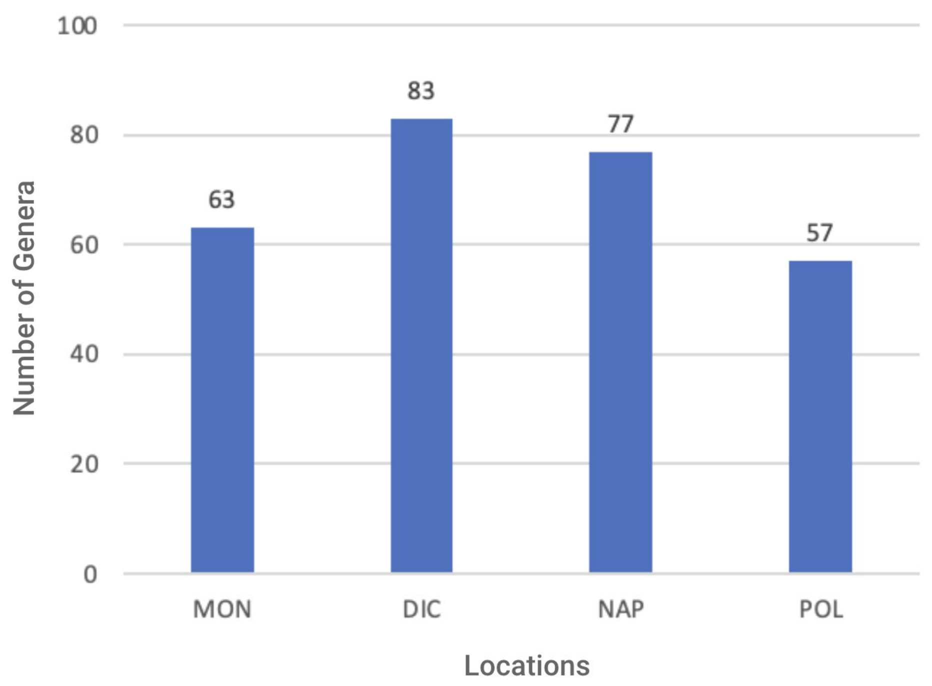

Figure S2. Diversity of genera within each location (MON, DIC, NAP, and POL). Of the 3 samples for each location, 2 of the 3 samples had the bacteria present to be considered within the microbiome profile.

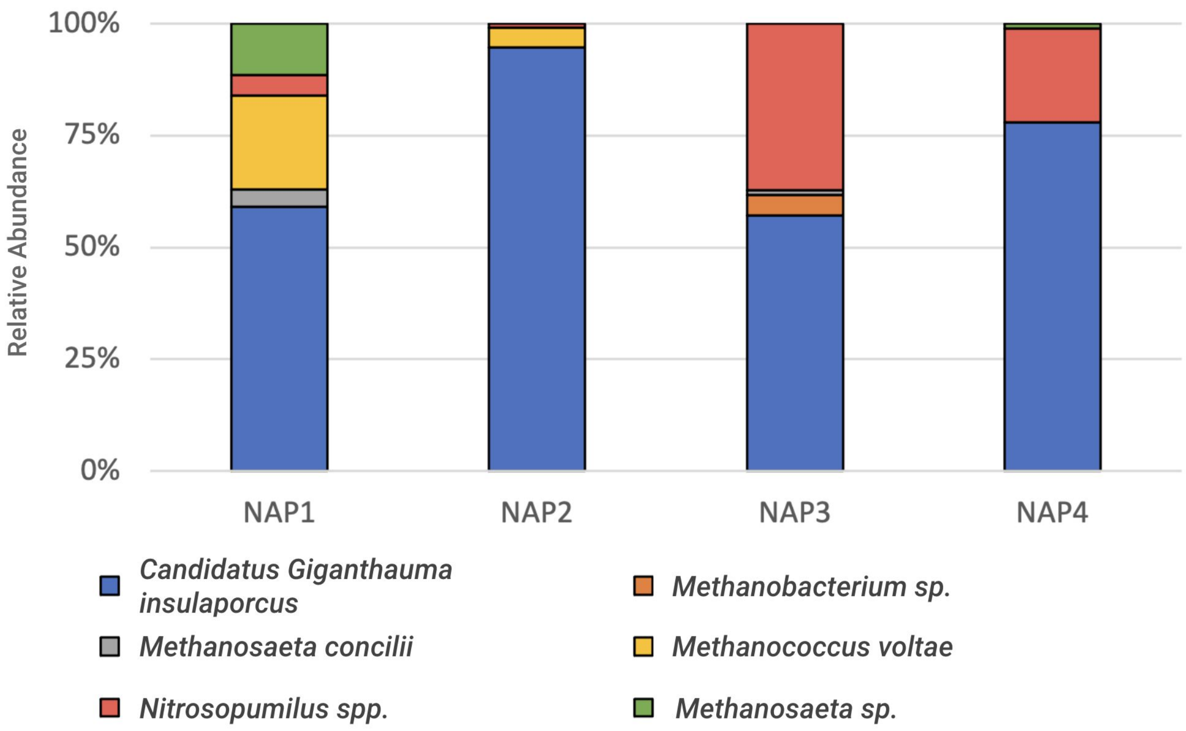

Figure S3. Bar Chart displaying the relative abundance of archaea species present in the four samples of egg capsules sequenced for archaeal DNA from Naples (NAP).

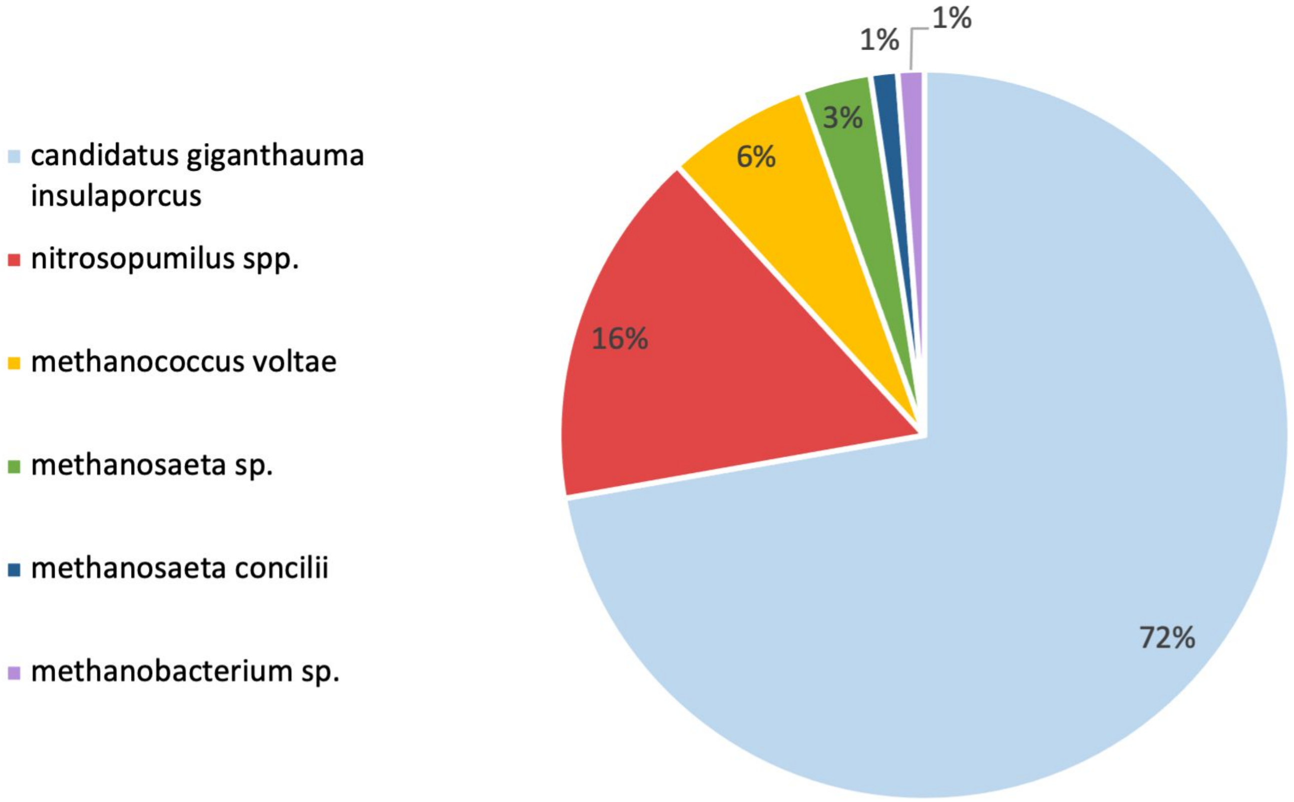

Figure S4. Pie chart of average relative abundance of archaea species within the egg capsules from Naples (NAP).

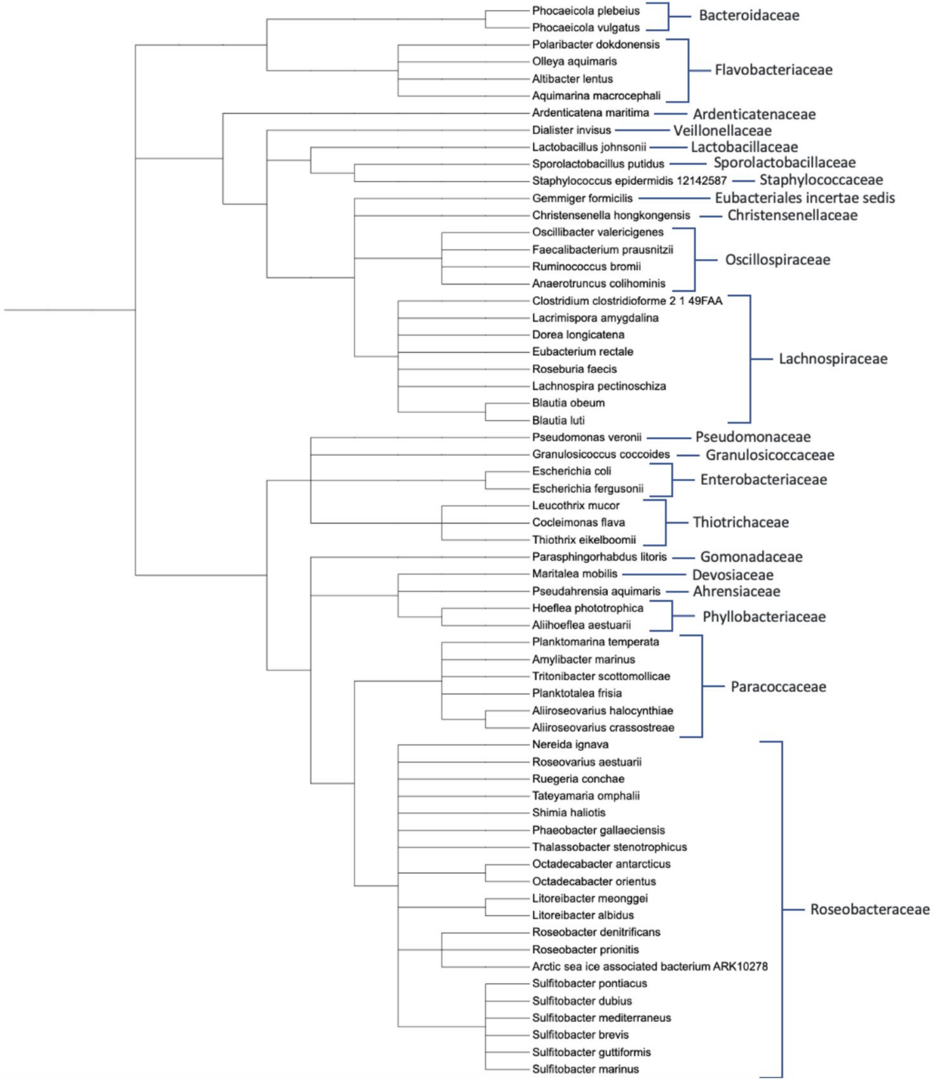

Figure S5. Phylogenetic tree of all identified core microbiome species and their taxonomic families. Tree was created using PhyloT and the NCBI Taxonomy Database.

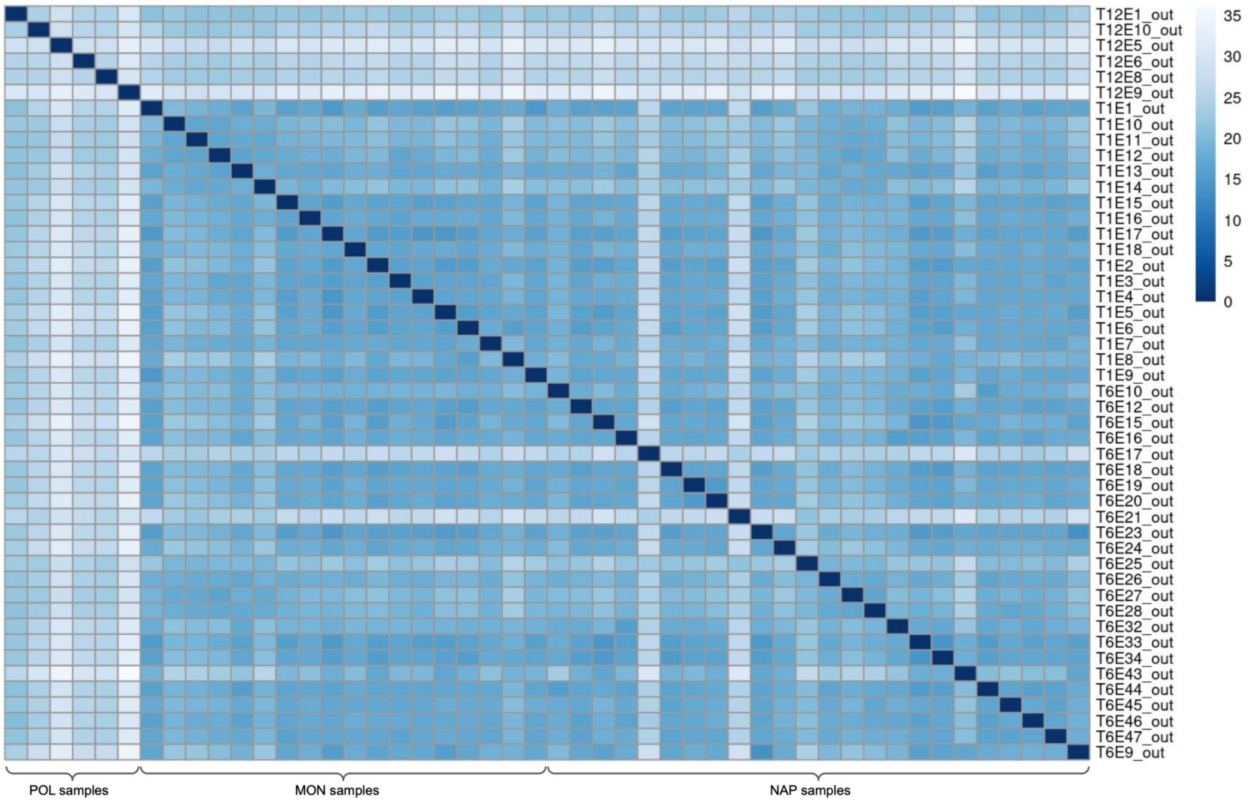

Figure S6. Correlation heatmap between sample gene expression in relation to the microbiome transcriptome. More similar sample gene expression is indicated by darker blue while more dissimilar sample gene expression is indicated by lighter blue.

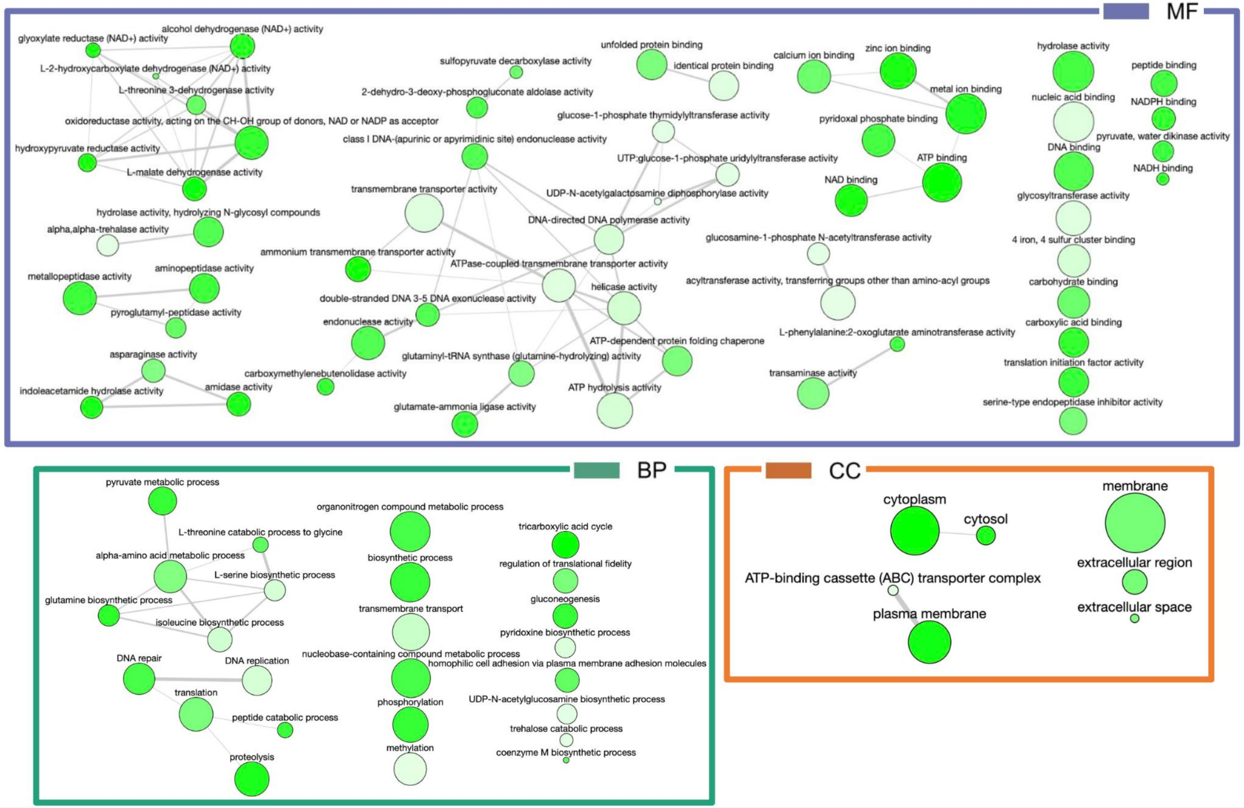

Figure S7. GO categorization (Biological process (BP), Molecular function (MF), Cellular component (CC)) of archaeal gene expression. Highly similar GO terms are linked by edges in the graph, where the line width indicates the degree of similarity. Color of the bubble indicates Log(TPM) with dark green being higher expression and light green being lower expression. Bubble size indicates the frequency of the GO term in the underlying GOA database.

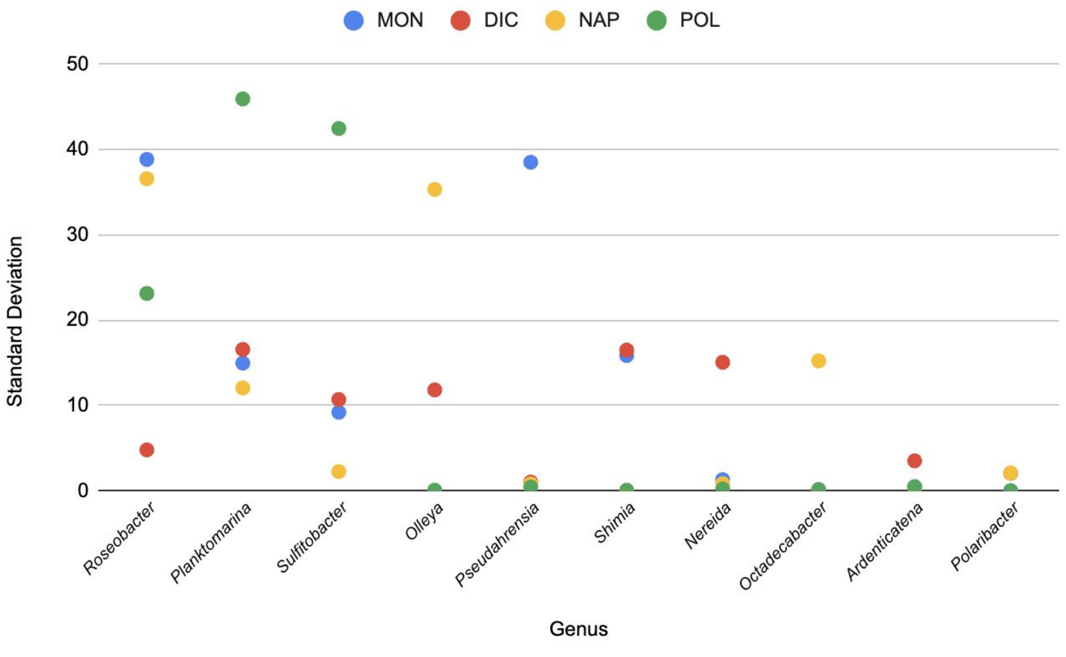

Figure S8. Comparison of variability within each sampling group. Standard deviation of top 10 most abundant genus within each location (MON, DIC, NAP, and POL).

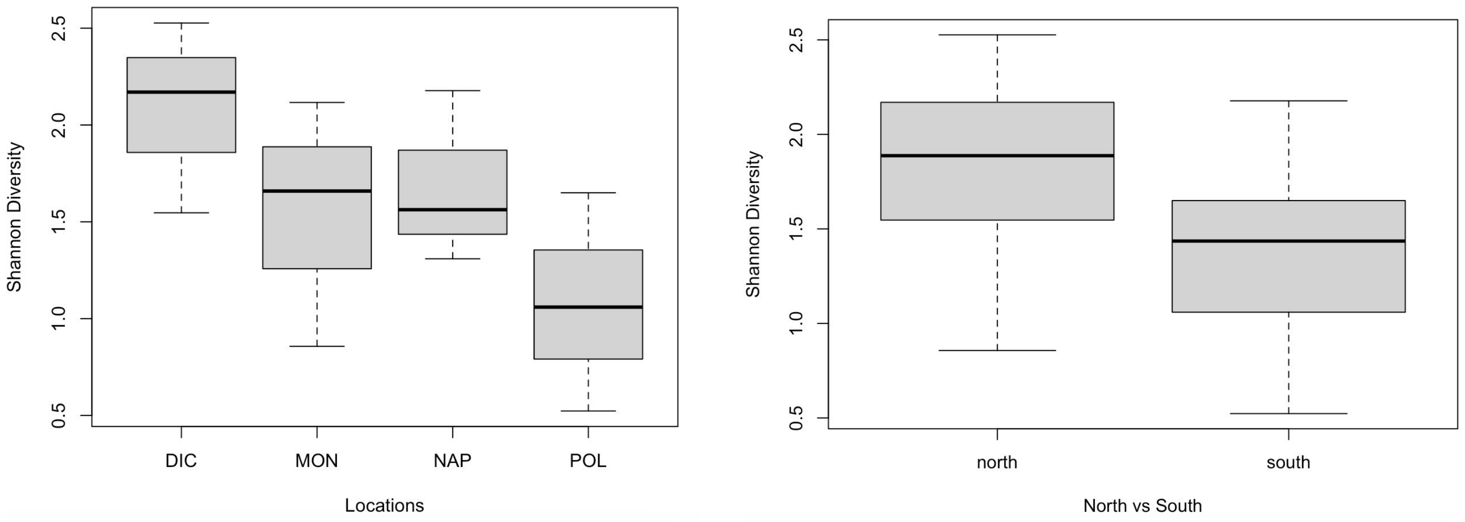

Figure S9. Box plot of Shannon diversity for each location and clustering North and South populations.
