# Supplementary material for: Microbiome composition and function within the Kellet’s whelk perivitelline fluid
Source: Microbiol Spectr. 2024 Feb 9;12(3):e03514-23. doi: 10.1128/spectrum.03514-23 (PMC10913743; doi:10.1128/spectrum.03514-23)
Supplement: Tables S1-S9 — Supplementary tables. [file spectrum.03514-23-s0002.pdf]

	MON		DIC		NAP		POL	
Rank	Phylum	% Abundance	Phylum	% Abundance	Phylum	% Abundance	Phylum	% Abundance
1	*Proteobacteria*	97.4	*Proteobacteria*	80.4	*Proteobacteria*	72.8	*Proteobacteria*	93.1
2	*Firmicutes*	1.43	*Bacteroidetes*	14.8	*Bacteroidetes*	22.7	*Bacteroidetes*	5.71
3	*Bacteroidetes*	0.79	*Chloroflexi*	2.8	*Firmicutes*	4.02	*Firmicutes*	0.81
4	*Chloroflexi*	0.28	*Firmicutes*	1.91	*Chloroflexi*	0.25	*Chloroflexi*	0.3
5	*Planctomycetes*	0.02	*Actinobacteria*	0.02	*Actinobacteria*	0.03	*Actinobacteria*	0.02
6			*Tenericutes*	0.01	*Planctomycetes*	0.03		
7					*Cyanobacteria*	0.02		

Table S1. Phyla ranking based on relative abundance across sampling locations.

**Location**	**Sample Label**	**Egg Capsule Collection Date**	**DNA Concentration (ng/µL)**
Monterey	MON1 (discarded)	05/30/2020	18.5
Monterey	MON2	06/09/2020	171
Monterey	MON3	06/18/2020	167
Monterey	MON4	05/30/2020	176
Diablo Canyon	DIC1	06/14/2020	38.7
Diablo Canyon	DIC2 (discarded)	06/18/2020	51.1
Diablo Canyon	DIC3	07/21/2020	156
Diablo Canyon	DIC4	06/09/2020	190
Naples	NAP1	06/14/2020	122
Naples	NAP2	06/18/2020	125
Naples	NAP3	07/07/2020	137
Naples	NAP4	06/14/2020	115
Point Loma	POL1	06/18/2020	147
Point Loma	POL2	07/08/2020	123
Point Loma	POL3 (discarded)	07/08/2020	100
Point Loma	POL4	07/08/2020	96

Table S2. DNA concentration and egg capsule collection date for each sample used for 16S rRNA gene sequencing.

Phylum	% Abundance	Class	% Abundance	Order	% Abundance	Family	% Abundance	Genus	% Abundance	Species	% Abundance
Proteobacteria	85.94	*Alphaproteobacteria*	83.67	*Rhodobacterales*	76.49	*Roseobacteraceae*	50.01	*Roseobacter*	27.53	*Planktomarina temperata*	25.9
Bacteroidetes	11.02	*Flavobacteriia*	10.72	*Flavobacteriales*	10.72	*Rhodobacteraceae*	26.49	*Planktomarina*	25.9	*Roseobacter prionitis*	16.2
Firmicutes	2.04	*Gammaproteobacteria*	2.12	*Hyphomicrobiales*	7.01	*Flavobacteriaceae*	10.61	*Sulfitobacter*	10.91	*Roseobacter arctic sea*	11.16
Chloroflexi	0.91	*Clostridia*	1.7	*Ardenticatenales*	0.87	*Ahrensiaceae*	6.72	*Olleya*	6.85	*Olleya aquimaris*	6.84
Actinobacteria	0.02	*Ardenticatenia*	0.87	*Thiotrichales*	0.78	*Ardenticatenaceae*	0.87	*Pseudahrensia*	6.71	*Pseudahrensia aquimaris*	6.71
		Bacilli	0.26	*Pseudomonadales*	0.54	*Thiotrichaceae*	0.78	*Shimia*	4.75	*Sulfitobacter brevis*	4.94
		Bacteroidia	0.15	*Enterobacterales*	0.21	*Lachnospiraceae*	0.74	*Nereida*	2.83	*Shimia haliotis*	4.72
		Saprospiria	0.13	*Cellvibrionales*	0.16	*Oscillospiraceae*	0.53	*Octadecabacter*	2.36	*Sulfitobacter pontiacus*	3.91
		Epsilonproteobacteria	0.08	*Bacillales*	0.15	*Pseudomonadaceae*	0.47	*Ardenticatena*	0.87	*Nereida ignava*	2.83
		Deltaproteobacteria	0.05	*Saprospirales*	0.13	*Eubacteriaceae*	0.28	*Polaribacter*	0.61	*Octadecabacter orientus*	2.22
		Negativicutes	0.03	*Bacteroidales*	0.12	*Enterobacteriaceae*	0.21	*Amylibacter*	0.53	*Sulfitobacter guttiformis*	1.51
				Lactobacillales	0.11	*Phyllobacteriaceae*	0.14	*Pseudomonas*	0.47	*Ardenticatena maritima*	0.87
				Sphingomonadales	0.09	*Lactobacillaceae*	0.1	*Roseovarius*	0.47	*Polaribacter dokdonensis*	0.61
				Campylobacterales	0.08	*Bacteroidaceae*	0.09	*Cocleimonas*	0.43	*Amylibacter rhodobacteraceae bacterium*	0.53
				Chromatiales	0.07	*Haliscomenobacteraceae*	0.08	*Litoreibacter*	0.36	*Cocleimonas flava*	0.43
				Myxococcales	0.03	*Staphylococcaceae*	0.07	*Thiothrix*	0.28	*Sulfitobacter marinus*	0.39
				Veillonellales	0.02	*Devosiaceae*	0.06	*Eubacterium*	0.28	*Roseovarius aestuarii*	0.35
				Maricaulales	0.02	*Sphingomonadaceae*	0.05	*Faecalibacterium*	0.28	*Thiothrix eikelboomii*	0.28
						Lewinellaceae	0.05	*Blautia*	0.27	*Faecalibacterium prausnitzii*	0.28
						Ruminococcaceae	0.04	*Escherichia*	0.21	*Litoreibacter albidus*	0.24
						Erythrobacteraceae	0.04	*Roseburia*	0.17	*Eubacterium rectale*	0.19
						Christensenellaceae	0.03	*Lachnoclostridium*	0.15	*Blautia ruminococcus obeum*	0.16
						Sporolactobacillaceae	0.03	*Ruegeria*	0.14	*Roseobacter denitrificans*	0.15
						Veillonellaceae	0.02	*Thalassobacter*	0.1	*Roseburia faecis*	0.15
						Ectothiorhodospiraceae	0.02	*Lactobacillus*	0.1	*Escherichia fergusonii*	0.15
						Robiginitomaculaceae	0.02	*Ruminococcus*	0.1	*Octadecabacter antarcticus*	0.13
						Granulosicoccaceae	0.01	*Bacteroides*	0.09	*Sulfitobacter mediterraneus*	0.1
								Loktanella	0.08	*Thalassobacter stenotrophicus*	0.1
								Oscillibacter	0.08	*Ruegeria scottomollicae*	0.09
								Staphylococcus	0.07	*Lachnoclostridium clostridium clostridioforme*	0.09
								Hoeflea	0.07	*Litoreibacter meonggei*	0.08
								Phaeobacter	0.06	*Lactobacillus johnsonii*	0.08
								Leucothrix	0.06	*Oscillibacter valericigenes*	0.08
								Lachnospira	0.05	*Hoeflea phototrophica*	0.07
								Pelagicola	0.05	*Phaeobacter gallaeciensis*	0.06
								Ruminiclostridium	0.05	*Leucothrix mucor*	0.06
								Planktotalea	0.05	*Escherichia coli*	0.06
								Lewinella	0.05	*Lachnospira pectinoschiza*	0.05
								Sphingorhabdus	0.04	*Roseovarius halocynthiae*	0.05
								Maritalea	0.04	*Planktotalea frisia*	0.05
								Gemmiger	0.04	*Ruminococcus bromii*	0.05
								Dorea	0.03	*Sphingorhabdus litoris*	0.04
								Aliihoeflea	0.03	*Gemmiger formicilis*	0.04
								Altibacter	0.03	*Blautia luti*	0.03
								Aquimarina	0.03	*Maritalea zhangella mobilis*	0.03
								Sporolactobacillus	0.03	*Aliihoeflea aestuarii*	0.03
								Coprococcus	0.03	*Dorea longicatena*	0.03
								Altererythrobacter	0.03	*Altibacter altuibacter lentus*	0.03
								Catabacter	0.02	*Pseudomonas veronii*	0.03
								Ulvibacter	0.02	*Sporolactobacillus putidus*	0.03
								Anaerotruncus	0.02	*Bacteroides vulgatus*	0.03
								Dialister	0.02	*Staphylococcus epidermidis*	0.03
								Granulosicoccus	0.01	*Aquimarina macrocephali*	0.03
								Tateyamaria	0.01	*Catabacter hongkongensis*	0.02
										Ruegeria conchae	0.02
										Anaerotruncus colihominis	0.02
										Bacteroides plebeius	0.02
										Dialister invisus	0.02
										Granulosicoccus coccoides	0.01
										Sulfitobacter dubius	0.01
										Lachnoclostridium clostridium amygdalinum	0.01
										Tateyamaria omphalii	0.01
										Roseovarius crassostreae	0.01

Table S3. All taxonomic groups their percent abundance identified within the core microbiome.

Organism	Total Genes	non DEGs	DEGs
Bacteria	11,561	11,542	19
Viruses	2,281	2,278	3
Archaea	301	301	0

Table S4. Total genes found in each microorganism transcriptome and number of differentially expressed genes from each organism between North and South populations.

Contig ID	Gene ID	Gene Name	% Identity	E-value	Bitscore	Origin
NODE_718895_length_463_cov_2.046154_g478567_i0	E0SCY1	Glycine betaine/choline transport system ATP-	78.571	5.73E-85	257	bacteria
NODE_1233323_length_252_cov_1.720670_g992587_i0	E0SCY2	Glycine betaine/choline transport system perm	87.952	6.72E-43	146	bacteria
NODE_1091121_length_283_cov_1.466667_g850388_i0	A3SK19	Dimethylsulfonioproprionate lyase DddP (DM	51.087	9.33E-25	98	bacteria
NODE_1042064_length_296_cov_2.475336_g801331_i0	Q5LLW7	3-methylmercaptopropionyl-CoA dehydrogena	56.122	1.53E-33	123	bacteria
NODE_936327_length_337_cov_1.003788_g695600_i0	Q5LRT0	3-methylmercaptopropionyl-CoA ligase (dmdI	53.947	1.75E-19	85	bacteria
NODE_1028550_length_301_cov_1.350877_g787818_i0	Q9K907	Bis(5'-nucleosyl)-tetraphosphatase PrpE (Ap4	42.667	1.08E-14	69	bacteria
NODE_826155_length_390_cov_2.343849_g585456_i0	Q3J6K9	Acrylyl-CoA reductase AcuI (Acryloyl-coenzy	57.317	2.76E-22	92	bacteria
NODE_437346_length_821_cov_2.528075_g217135_i0	A4YI89	3-hydroxypropionyl-coenzyme A dehydratase	33.908	2.90E-29	110	archaea
NODE_624412_length_551_cov_0.805439_g386099_i0	Q975C8	Acryloyl-coenzyme A reductase (Acryloyl-Co	35.398	7.64E-11	59	archaea

Table S5. Microbiome transcriptome contig BLAST results to DMSP related proteins.

Inhibit Host Immune Response					
Contig ID	Gene ID	Gene Name	% Identity	E-value	Bitscore
NODE_52560_length_3938_cov_14092.971798_g13429_i0	D0ZPH9	E3 ubiquitin-protein ligase SspH2	25.146	3.01E-07	58.2
NODE_102930_length_2730_cov_602.579601_g8106_i5	Q8ZQQ2	E3 ubiquitin-protein ligase SlrP	26.897	6.17E-07	56.2
NODE_875141_length_364_cov_2.202749_g634426_i0	Q6MWY2	Uncharacterized PPE family protein PPE54	37.5	2.12E-06	47.8
NODE_39730_length_4481_cov_153.611842_g10250_i0	P29129	E3 ubiquitin-protein ligase ICP0	40	7.09E-06	49.7
NODE_1347732_length_227_cov_3.000000_g1106996_i0	P9WJN1	Mycothiol S-conjugate amidase	74.51	9.98E-21	84.7

Communication with Host					
Contig ID	Gene ID	Gene Name	% Identity	E-value	Bitscore
NODE_505481_length_700_cov_4.926635_g275494_i0	P14727	Avirulence protein AvrBs3 (TAL effector protein AvrBs3)	24	0.00000154	51.2
NODE_682201_length_494_cov_40.931116_g442301_i0	B2SU53	TAL effector protein PthXo1 (Avirulence protein PthXo1)	28.659	0.00000221	49.3
NODE_485427_length_732_cov_4.880121_g257817_i0	Q56830	Avirulence protein AvrXa10 (TAL effector protein AvrXa10)	27.041	9.05E-10	61.2
NODE_1031237_length_300_cov_1.559471_g790505_i0	Q79FI9	PPE family protein PPE34	37.037	0.00000762	45.4
NODE_515521_length_685_cov_2.215686_g284470_i0	P68924	Protein rexA	100	3.60E-102	296.0
NODE_1236632_length_251_cov_2.949438_g995896_i0	O30807	NAD-dependent malic enzyme (NAD-ME)	67.647	1.75E-07	49.3
NODE_1198061_length_259_cov_2.069892_g957325_i0	Q52978	Probable K(+)/H(+) antiporter subunit A/B	54.878	5.04E-15	70.9
NODE_1233785_length_252_cov_1.703911_g993049_i0	Q52981	Probable K(+)/H(+) antiporter subunit D	59.036	1.02E-25	100.0
NODE_1283039_length_245_cov_1.343023_g1042303_i0	Q1MDE9	Leu/Ile/Val-binding protein BraC3	41.429	9.99E-07	47.0
NODE_562092_length_621_cov_4.364964_g327142_i0	Q986R8	C4-dicarboxylate transport protein 2	80.667	2.16E-80	248.0
NODE_989601_length_315_cov_1.909091_g748872_i0	P22983	Pyruvate, phosphate dikinase	62.857	1.78E-40	144.0
NODE_812710_length_398_cov_1.726154_g572023_i0	P24290	Probable transcriptional activator (ORF-240)	27.5	4.54E-07	49.7
NODE_1266808_length_247_cov_1.770115_g1026072_i0	Q59207	Nitrogen fixation protein FixI (E1-E2 type cation ATPase FixI)	90.244	1.45E-37	134.0
NODE_237774_length_1486_cov_3.292286_g82278_i0	O33533	Nitrogen fixation protein FixI (E1-E2 type cation ATPase FixI)	45.687	1.24E-52	192.0
NODE_410582_length_878_cov_1.925466_g195596_i0	P18398	Nitrogen fixation protein FixI (E1-E2 type cation ATPase FixI)	58.419	7.50E-101	313.0

Communication Between Microbes					
Contig ID	Gene ID	Gene Name	% Identity	E-value	Bitscore
NODE_1260008_length_248_cov_1.760000_g1019272_i0	P0AFS6	AI-2 transport protein TqsA	100	1.69E-29	109.0
NODE_1304230_length_242_cov_1.822485_g1063494_i0	Q0T1B8	S-ribosylhomocysteine lyase (LuxS)	100	9.82E-56	171.0
NODE_1291426_length_244_cov_0.900585_g1050690_i0	D5CBA0	16S rRNA endonuclease CdiA (rRNase CdiA)	44.898	0.00000398	45.4
NODE_767263_length_427_cov_1.087571_g526675_i0	I1WVY3	tRNA nuclease CdiA-2 (tRNase CdiA-2)	41.007	4.85E-17	79.3
NODE_711230_length_469_cov_3.851010_g470962_i0	Q9I194	Acyl-homoserine lactone acylase PvdQ (AHL acylase PvdQ)	44.872	5.16E-34	128.0
NODE_730411_length_454_cov_1.532808_g489999_i0	E5KK10	Methanogenesis regulatory histidine kinase FilI (AHL synthase)	31.333	9.91E-15	69.3
NODE_1027888_length_301_cov_1.956140_g787156_i0	G3XD24	Methyl-accepting chemotaxis protein PctA	47.191	1.56E-21	90.1
NODE_844403_length_380_cov_2.006515_g603692_i0	Q9I0I4	Methyl-accepting chemotaxis protein TlpQ (TlpQ chemoreceptor)	38.318	9.51E-18	80.5

Table S6. Microbiome transcriptome contig BLAST results to host interactions and symbiosis.

Polyketide Antibiotics					
**Contig ID**	**Gene ID**	**Gene Name**	**% Identity**	**E-value**	**Bitscore**
NODE_1303549_length_242_cov_1.822485_g1062813_i0	P40806	Polyketide synthase (PksJ)	66.25	2.17E-32	120
NODE_1218360_length_255_cov_1.692308_g977624_i0	Q2T4N1	Polyketide synthase (ThaG)	53.012	9.56E-22	90.1
NODE_958144_length_328_cov_1.203922_g717416_i0	Q84HC6	Neocarzinostatin naphthoate synthase (NcsB)	32.692	9.37E-09	53.9

Etamycin					
**Contig ID**	**Gene ID**	**Gene Name**	**% Identity**	**E-value**	**Bitscore**
NODE_250545_length_1419_cov_129.500000_g15903_i1	R9UTQ8	L-proline trans-4-hydroxylase (P4H)	30.531	2.59E-23	101

Midecamycin					
**Contig ID**	**Gene ID**	**Gene Name**	**% Identity**	**E-value**	**Bitscore**
NODE_205957_length_1672_cov_4877.799875_g42603_i5	Q00719	O-methyltransferase (MdmC)	40.777	3.97E-14	74.3
NODE_1364693_length_212_cov_4.431655_g1123957_i0	Q59523	Mycinamicin IV hydroxylase/epoxidase (MycG)	37.681	0.00000348	45.1

Rebeccamycin					
**Contig ID**	**Gene ID**	**Gene Name**	**% Identity**	**E-value**	**Bitscore**
NODE_761131_length_431_cov_2.572626_g520562_i0	Q8KHE4	4'-demethylrebeccamycin synthase (RebG)	49.296	2.01E-34	126

Pyrrolnitrin					
**Contig ID**	**Gene ID**	**Gene Name**	**% Identity**	**E-value**	**Bitscore**
NODE_1281888_length_245_cov_1.790698_g1041152_i0	P95480	Flavin-dependent tryptophan halogenase (PrnA)	40.26	1.67E-11	60.5
NODE_345788_length_1048_cov_3.645128_g147783_i0	P25026	Non-heme chloroperoxidase (CpoP)	75.706	3.17E-89	271

Gramicidin					
**Contig ID**	**Gene ID**	**Gene Name**	**% Identity**	**E-value**	**Bitscore**
NODE_276125_length_1301_cov_3.438925_g103308_i0	Q70LM5	Linear gramicidin synthase subunit C (LgrC)	52.358	6.97E-123	396
NODE_1034189_length_299_cov_1.438053_g793457_i0	Q70LM6	Linear gramicidin synthase subunit B (LgrB)	48.98	5.06E-22	91.3
NODE_1142406_length_271_cov_1.944444_g901671_i0	Q70LM4	Linear gramicidin synthase subunit D (LgrD)	51.948	1.98E-17	77.8

Oxytetracycline					
**Contig ID**	**Gene ID**	**Gene Name**	**% Identity**	**E-value**	**Bitscore**
NODE_999760_length_311_cov_2.264706_g759030_i0	L8EUQ6	12-dehydrotetracycline 5-monooxygenase (OxyS)	58.182	1.08E-14	70.5
NODE_1306519_length_242_cov_0.911243_g1065783_i0	Q3S8R0	6-methylpretetramide 4-monooxygenase (OxyE)	55.932	2.91E-12	62.8

Plipastatin					
**Contig ID**	**Gene ID**	**Gene Name**	**% Identity**	**E-value**	**Bitscore**
NODE_1255620_length_249_cov_0.875000_g1014884_i0	O31827	Plipastatin synthase subunit E (PpsE)	35.616	6.50E-08	50.4
NODE_1146719_length_270_cov_2.045685_g905983_i0	P39846	Plipastatin synthase subunit B (PpsB)	40	3.87E-07	48.5

Table S7. Microbiome transcriptome contig BLAST results to antibiotic biosynthesis.

Contig ID	Gene ID	Gene Name	% Identity	E-value	Bitscore	Organism
NODE_251686_length_1414_cov_2.871738_g89587_i0	P03708	Terminase, large subunit (DNA-packaging protein A)	100	0	990	Escherichia phage lambda (Bacteriophage lambda)
NODE_288303_length_1250_cov_1.480884_g110510_i0	P03710	Portal protein B (GpB) (Minor capsid protein B)	100	0	624	Escherichia phage lambda (Bacteriophage lambda)
NODE_396311_length_911_cov_2.995227_g184505_i0	P03749	Tip attachment protein J (Central tail fiber)	99.634	0	563	Escherichia phage lambda (Bacteriophage lambda)
NODE_544567_length_644_cov_1.618214_g310839_i0	P03753	Protein ea31	100	7.07E-165	455	Escherichia phage lambda (Bacteriophage lambda)
NODE_361943_length_1001_cov_3.101293_g159058_i0	P03688	Replication protein O	99.543	1.60E-161	453	Escherichia phage lambda (Bacteriophage lambda)
NODE_297658_length_1213_cov_2.607895_g116158_i0	P03697	Exonuclease	100	7.79E-157	440	Escherichia phage lambda (Bacteriophage lambda)
NODE_318711_length_1136_cov_2.653810_g129551_i0	P03729	Tail tip assembly protein K (Probable endopeptidase)	100	1.91E-141	399	Escherichia phage lambda (Bacteriophage lambda)
NODE_190932_length_1778_cov_4.013490_g59991_i0	P03701	Outer membrane protein lom (ORF 206A)	100	4.30E-132	384	Escherichia phage lambda (Bacteriophage lambda)
NODE_502476_length_705_cov_1.898734_g272830_i0	P03754	Protein ea59	100	7.64E-130	375	Escherichia phage lambda (Bacteriophage lambda)
NODE_263458_length_1358_cov_1.866148_g96090_i0	P03770	Protein ninG	100	5.66E-117	340	Escherichia phage lambda (Bacteriophage lambda)
NODE_311122_length_1163_cov_4.355963_g124620_i0	P03706	Endolysin (EC 4.2.2.n2) (Lysis protein) (Lysozyme) (Transglycosylase)	100	3.46E-114	328	Escherichia phage lambda (Bacteriophage lambda)
NODE_469260_length_760_cov_2.727802_g243845_i0	P03764	Tail fiber protein (stf) (Gene product 27) (gp27)	98.969	3.60E-111	300	Escherichia phage lambda (Bacteriophage lambda)
NODE_515521_length_685_cov_2.215686_g284470_i0	P68924	Protein rexA	100	3.60E-102	296	Escherichia phage lambda (Bacteriophage lambda)
NODE_228534_length_1537_cov_4.150273_g77662_i0	P03765	Protein ninB	100	3.66E-101	300	Escherichia phage lambda (Bacteriophage lambda)
NODE_783587_length_416_cov_2.244898_g542951_i0	P03762	Superinfection exclusion protein B	100	4.92E-101	286	Escherichia phage lambda (Bacteriophage lambda)
NODE_679394_length_497_cov_2.820755_g439542_i0	P03707	Terminase small subunit (gpNu1)	100	2.19E-99	283	Escherichia phage lambda (Bacteriophage lambda)
NODE_494900_length_717_cov_2.732919_g266153_i0	P27078	Integrase	100	9.52E-87	260	Enterobacteria phage 434 (Bacteriophage 434)
NODE_916084_length_345_cov_3.136029_g675358_i0	P03756	Protein ea22	100	4.65E-82	237	Escherichia phage lambda (Bacteriophage lambda)
NODE_937963_length_336_cov_1.901141_g697236_i0	P03738	Tail tip protein L	100	9.74E-80	233	Escherichia phage lambda (Bacteriophage lambda)
NODE_793013_length_410_cov_2.000000_g552349_i0	P03737	Tail tip protein M	100	5.54E-78	225	Escherichia phage lambda (Bacteriophage lambda)
NODE_500575_length_708_cov_1.573228_g271173_i0	P03733	Tail tube protein (TTP) (Gene product V) (gpV) (Major tail protein V)	100	7.17E-77	231	Escherichia phage lambda (Bacteriophage lambda)
NODE_1055624_length_292_cov_1.406393_g814891_i0	P03713	Major capsid protein (Gene product E) (gpE) (Major head protein)	100	1.45E-65	199	Escherichia phage lambda (Bacteriophage lambda)
NODE_770170_length_425_cov_1.531250_g529572_i0	P51767	Baseplate protein J (Gene J protein) (GpJ)	67.376	2.69E-64	197	Escherichia phage P2 (Bacteriophage P2)
NODE_758077_length_433_cov_3.225000_g517518_i0	P03732	Tail tube terminator protein (TrP) (Gene product U) (gpU)	100	4.29E-64	191	Escherichia phage lambda (Bacteriophage lambda)
NODE_801175_length_405_cov_1.789157_g560496_i0	P03755	Protein ea8.5	100	5.59E-58	174	Escherichia phage lambda (Bacteriophage lambda)
NODE_446192_length_803_cov_4.104110_g224426_i0	P03658	Gene 1 protein (G1P)	46.89	5.81E-58	188	Salmonella phage IKe (Bacteriophage IKe)
NODE_1081518_length_285_cov_2.179245_g840785_i0	P03736	Tape measure protein (TMP) (Gene product H) (gpH)	100	2.43E-57	187	Escherichia phage lambda (Bacteriophage lambda)
NODE_704440_length_475_cov_1.982587_g464250_i0	P03698	Recombination protein bet	100	6.35E-55	172	Escherichia phage lambda (Bacteriophage lambda)
NODE_702925_length_476_cov_3.796526_g462749_i0	P03740	Tail fiber assembly protein	100	6.82E-49	155	Escherichia phage lambda (Bacteriophage lambda)
NODE_1093265_length_282_cov_3.602871_g852532_i0	O22010	Excisionase	81.111	1.70E-48	150	Shigella phage SfV (Shigella flexneri bacteriophage V) (Bacteriophage SfV)
NODE_1130963_length_274_cov_1.149254_g890228_i0	P03711	Capsid assembly protease C (GPC) (Minor capsid protein C)	100	1.29E-47	155	Escherichia phage lambda (Bacteriophage lambda)
NODE_625082_length_550_cov_3.006289_g386726_i0	P03758	Protein kil	100	4.29E-47	148	Escherichia phage lambda (Bacteriophage lambda)
NODE_1255831_length_249_cov_0.875000_g1015095_i0	P03735	Tail assembly protein GT (Gene product 15) (gp15)	100	1.50E-41	136	Escherichia phage lambda (Bacteriophage lambda)
NODE_1375839_length_200_cov_7.275591_g1135103_i0	P00573	T7 RNA polymerase (DNA-directed RNA polymerase)	100	3.06E-41	141	Escherichia phage T7 (Bacteriophage T7)
NODE_540238_length_650_cov_1.334489_g306876_i0	P03773	Putative uncharacterized protein ORF64	100	3.54E-38	126	Escherichia phage lambda (Bacteriophage lambda)
NODE_864760_length_369_cov_3.347973_g624046_i0	P03767	Protein ninD	98.246	3.30E-36	117	Escherichia phage lambda (Bacteriophage lambda)
NODE_950887_length_331_cov_1.193798_g710160_i0	P03731	Tail completion protein Z (Gene product Z) (GpZ)	100	2.51E-35	119	Escherichia phage lambda (Bacteriophage lambda)
NODE_1300691_length_243_cov_0.900000_g1059955_i0	P03042	Transcriptional activator II	100	3.68E-35	114	Escherichia phage lambda (Bacteriophage lambda)
NODE_630994_length_544_cov_2.938429_g392407_i0	P03045	Antitermination protein N (Regulatory protein N) (PN)	100	1.69E-32	111	Escherichia phage lambda (Bacteriophage lambda)
NODE_1020003_length_304_cov_1.303030_g779272_i0	P10311	DNA-invertase (Site-specific recombinase)	54.545	3.97E-32	110	Escherichia phage P1 (Bacteriophage P1)
NODE_829489_length_388_cov_2.933333_g588786_i0	P03015	Serine recombinase gin (Gene product 53) (gp53)	54.902	3.99E-31	108	Escherichia phage Mu (Bacteriophage Mu)
NODE_549705_length_637_cov_2.306738_g315589_i0	P08320	Integrase	32.836	1.92E-27	107	Enterobacteria phage P4 (Bacteriophage P4)
NODE_1069337_length_288_cov_2.148837_g828604_i0	P51739	Uncharacterized 58.7 kDa protein in lys 3'region (ORF35)	45.833	1.25E-26	100	Haemophilus phage HP1 (strain HP1c1) (Bacteriophage HP1)
NODE_1259416_length_248_cov_1.805714_g1018680_i0	P03766	Uncharacterized nin region protein ORF290	55.294	1.74E-24	92	Escherichia phage lambda (Bacteriophage lambda)
NODE_977812_length_320_cov_0.882591_g737084_i0	Q9XJQ4	Protein ninG	50	1.79E-24	91.3	Enterobacteria phage P21 (Bacteriophage 21) (Bacteriophage P21)
NODE_1239776_length_251_cov_1.297753_g999040_i0	P49861	Major capsid protein (Gene product 5) (gp5)	49.398	6.77E-19	78.2	Enterobacteria phage HK97 (Bacteriophage HK97)
NODE_823968_length_391_cov_3.632075_g583271_i0	P26814	Lipoprotein bor	100	5.51E-17	70.1	Escherichia phage lambda (Bacteriophage lambda)
NODE_1116593_length_277_cov_1.509804_g875858_i0	Q9T1Q3	Probable DNA polymerase (P45)	43.182	6.92E-17	73.2	Acyrthosiphon pisum secondary endosymbiont phage 1 (Bacteriophage APSE-1)
NODE_885781_length_359_cov_1.874126_g645064_i0	O64203	Endolysin A (Gene 10 protein) (Gp10)	44.706	2.01E-15	70.1	Mycobacterium phage D29 (Mycobacteriophage D29)
NODE_1314834_length_241_cov_0.916667_g1074098_i0	Q01435	Uncharacterized 7.2 kDa protein in Gp39-comCA intergenic region	56.863	7.27E-14	59.3	Enterobacteria phage T4 (Bacteriophage T4)
NODE_1313991_length_241_cov_0.916667_g1073255_i0	P57020	Antitermination protein Q	40.506	3.96E-13	60.8	Salmonella phage P22 (Bacteriophage P22)
NODE_1162318_length_267_cov_1.298969_g921582_i0	O64333	Tail tip assembly protein K (Gene product 19) (gp19)	60.976	7.97E-13	60.8	Escherichia phage N15 (Bacteriophage N15)
NODE_1269522_length_247_cov_0.885057_g1028786_i0	Q0PDK7	Tail tape measure protein gp18 (Gene product 18) (gp18)	38.028	8.41E-13	61.2	Bacillus phage SPP1 (Bacteriophage SPP1)
NODE_1202438_length_258_cov_2.497297_g961702_i0	P18771	Long-tail fiber proximal subunit (Gene product 34) (gp34)	38.462	2.95E-12	59.7	Enterobacteria phage T4 (Bacteriophage T4)
NODE_1306711_length_242_cov_0.911243_g1065975_i0	D6RRG1	Major capsid protein (Gene product 4) (gp4) (Major head protein)	57.895	2.04E-11	57	Pseudomonas phage KPP10 (Bacteriophage KPP10)
NODE_320161_length_1131_cov_3.546314_g130530_i0	P06155	Integrase	27.5	1.26E-10	61.6	Enterobacteria phage phi80 (Bacteriophage phi-80)
NODE_1349873_length_226_cov_3.405229_g1109137_i0	P00814	Deoxycytidylate deaminase (EC 3.5.4.12) (dCMP deaminase)	43.636	1.12E-09	51.2	Enterobacteria phage T2 (Bacteriophage T2)
NODE_771657_length_424_cov_1.481481_g531054_i0	O64253	Gene 61 protein (Gp61)	32.5	1.37E-09	52	Mycobacterium phage D29 (Mycobacteriophage D29)
NODE_1260358_length_248_cov_1.714286_g1019622_i0	P68652	Protein ninB	42.424	2.73E-09	49.7	Enterobacteria phage P21 (Bacteriophage 21) (Bacteriophage P21)
NODE_1150269_length_269_cov_3.142857_g909533_i0	P25478	Capsid assembly scaffolding protein (Gene product O) (GpO)	41.333	3.02E-09	51.2	Escherichia phage P2 (Bacteriophage P2)
NODE_14224_length_6641_cov_3.735079_g3938_i0	Q9T1V2	Baseplate protein gp47 (Gene product 47) (gp47) (gpW)	43.421	3.89E-09	60.5	Escherichia phage Mu (Bacteriophage Mu)
NODE_840596_length_382_cov_2.291262_g599885_i0	P12552	Uncharacterized protein ORF88 (Putative DNA-binding protein)	44.681	1.19E-08	48.1	Enterobacteria phage P4 (Bacteriophage P4)
NODE_536131_length_655_cov_8.024055_g303092_i0	Q9T1Q1	Putative protein p47	44.776	1.21E-08	52.4	Acyrthosiphon pisum secondary endosymbiont phage 1 (Bacteriophage APSE-1)
NODE_1112553_length_278_cov_1.502439_g871819_i0	Q05280	Gene 66 protein (Gp66)	43.333	1.49E-08	48.9	Mycobacterium phage L5 (Mycobacteriophage L5)
NODE_1122104_length_276_cov_1.137931_g881369_i0	P25479	Terminase, ATPase subunit (GpP)	58.14	0.00000012	47	Escherichia phage P2 (Bacteriophage P2)
NODE_1214260_length_256_cov_1.469945_g973524_i0	A0MZE7	Non-contractile tail sheath (Gene product 65) (Gp65)	50	1.59E-07	46.2	Enterobacteria phage N4 (Bacteriophage N4)
NODE_1106101_length_279_cov_3.067961_g865367_i0	O64046	Probable tape measure protein (TMP) (Transglycosylase)	39.535	1.91E-07	46.2	Bacillus phage SPbeta (Bacteriophage SP-beta)
NODE_1313537_length_241_cov_0.916667_g1072801_i0	P03730	Tail tip assembly protein I	36.17	2.29E-07	45.4	Escherichia phage lambda (Bacteriophage lambda)
NODE_1165654_length_266_cov_1.658031_g924918_i0	P20345	Pre-neck appendage protein (Gene product 12) (gp12) (NP1)	40.385	2.39E-07	45.8	Bacillus phage phi29 (Bacteriophage phi-29)
NODE_1202506_length_258_cov_2.459459_g961770_i0	Q6WHG9	Protein Gp5	41.379	2.63E-07	45.8	Vibrio phage KVP40 (KVP40) (Bacteriophage KVP40)
NODE_1062354_length_290_cov_1.419355_g821621_i0	P11187	Endolysin (EC 3.2.1.17) (Lysis protein) (Lysozyme) (Protein p15)	35.556	6.39E-07	44.7	Bacillus phage phi29 (Bacteriophage phi-29)
NODE_861556_length_371_cov_1.859060_g620842_i0	P03796	Protein 7.7 (Gene product 7.7) (Gp7.7)	31.624	0.00000167	43.5	Escherichia phage T7 (Bacteriophage T7)
NODE_1129972_length_274_cov_1.532338_g889237_i0	Q37906	Repressor protein CI	45.455	0.00000235	43.1	Pseudomonas phage D3 (Bacteriophage D3)
NODE_1331402_length_234_cov_2.869565_g1090666_i0	P13772	ImmF control region 10 kDa protein	40.385	0.00000236	40.8	Bacillus phage phi105 (Bacteriophage phi-105)
NODE_651435_length_524_cov_1.718404_g412213_i0	P04890	Integrase	29.167	0.00000286	45.4	Salmonella phage P22 (Bacteriophage P22)
NODE_1037568_length_298_cov_1.026667_g796835_i0	P59217	Putative terminase large subunit	30	0.00000286	43.1	Shigella phage SfV (Shigella flexneri bacteriophage V)
NODE_492611_length_720_cov_586.174652_g264153_i0	P13299	Intron-associated endonuclease 1 (I-TevI) (IRF protein)	25	0.00000361	46.2	Enterobacteria phage T4 (Bacteriophage T4)
NODE_1100255_length_281_cov_1.221154_g859522_i0	P32277	RNA ligase 2 (Rnl2)	41.304	0.00000653	42	Enterobacteria phage T4 (Bacteriophage T4)

Table S8. Microbiome transcriptome contig BLAST results to bacteriophage gene expression.

Comparsion	diff	lwr	upr	p adj
MON-DIC	-0.5369	-1.9508	0.8768	0.6345
NAP-DIC	-0.3979	-1.8117	1.0158	0.8047
POL-DIC	-1.0034	-2.4172	0.4103	0.1837
NAP-MON	0.1390	-1.2747	1.5528	0.9884
POL-MON	-0.4664	-1.8802	0.9473	0.7233
POL-NAP	-0.6055	-2.0193	0.8083	0.5484
SOUTH-NORTH	-0.4322	-1.1736	0.3092	0.2231

Table S9. ANOVA test comparing shannon diversity across sampling locations.
